# Adverse renal effects of NLRP3 inflammasome inhibition by MCC950 in an interventional model of diabetic kidney disease

**DOI:** 10.1042/CS20210865

**Published:** 2022-01-20

**Authors:** Jakob A. Østergaard, Jay C. Jha, Arpeeta Sharma, Aozhi Dai, Judy S.Y. Choi, Judy B. de Haan, Mark E. Cooper, Karin Jandeleit-Dahm

**Affiliations:** 1 Department of Diabetes, Central Clinical School, Monash University, Melbourne, Victoria, Australia; 2 Steno Diabetes Center Aarhus, Aarhus University Hospital, Aarhus, Denmark; 3 Department of Endocrinology and Internal Medicine, Aarhus University Hospital, Aarhus, Denmark; 4 Baker Heart and Diabetes Institute, Oxidative Stress Laboratory, Melbourne, Victoria, Australia; 5 Department of Immunology and Pathology, Central Clinical School, Monash University, Melbourne, Victoria, Australia; 6 Department of Cardiometabolic Health, Melbourne University, Melbourne, Victoria, Australia; 7 German Diabetes Centre, Leibniz Centre for Diabetes Research at the Heinrich Heine University, Duesseldorf, Germany

**Keywords:** diabetic nephropathy, inflammation, MCC950, NLRP3 inflammasome, oxidative stress

## Abstract

Activation of nucleotide-binding oligomerization domain-like receptor pyrin domain containing 3 (NLRP3) inflammasome has been reported in diabetic complications including diabetic kidney disease (DKD). However, it remains unknown if NLRP3 inhibition is renoprotective in a clinically relevant interventional approach with established DKD. We therefore examined the effect of the NLRP3-specific inhibitor MCC950 in streptozotocin-induced diabetic mice to measure the impact of NLRP3 inhibition on renal inflammation and associated pathology in DKD. We identified an adverse effect of MCC950 on renal pathology in diabetic animals. Indeed, MCC950-treated diabetic animals showed increased renal inflammation and macrophage infiltration in association with enhanced oxidative stress as well as increased mesangial expansion and glomerulosclerosis when compared with vehicle-treated diabetic animals. Inhibition of the inflammasome by MCC950 in diabetic mice led to renal up-regulation of markers of inflammation (*Il1*β, *Il18* and *Mcp1*), fibrosis (*Col1, Col4*, *Fn1, α-SMA*, *Ctgf* and* Tgfβ1*) and oxidative stress (*Nox2*, *Nox4* and nitrotyrosine*).* In addition, enhanced glomerular accumulation of pro-inflammatory CD68 positive cells and pro-oxidant factor nitrotyrosine was identified in the MCC950-treated diabetic compared with vehicle-treated diabetic animals. Collectively, in this interventional model of established DKD, NLRP3 inhibition with MCC950 did not show renoprotective effects in diabetic mice. On the contrary, diabetic mice treated with MCC950 exhibited adverse renal effects particularly enhanced renal inflammation and injury including mesangial expansion and glomerulosclerosis.

## Introduction

Diabetic kidney disease (DKD) develops in up to 40% of diabetic patients and remains the major cause of end-stage renal disease worldwide contributing to increased morbidity and mortality with current therapies only slowing its progression at best [1]. It is therefore of interest that increasing evidence supports the concept that nucleotide-binding oligomerization domain-like receptor pyrin domain containing 3 (NLRP3) inflammasome activation contributes to acute and chronic kidney disease including DKD [2–5]. For example, diabetic *Nlrp3*^−/−^ mice have ameliorated urinary albumin excretion and mesangial expansion compared with wildtype diabetic animals [5].

However, the inflammasome axis is also central for tissue homeostasis and thus blockade may potentially have adverse effects. Indeed, inhibition at the terminal level of the NLRP3 inflammasome axis, IL-1β, with canakinumab (a monoclonal IL-1β antibody) reduced cardiovascular events in the recent CANTOS trial [6], but disturbingly also led to a loss in eGFR in subjects with chronic kidney disease [7]. Therefore, with the wider application of strategies targeting the inflammasome axis, it is critical to define the renal impact of such approaches in order to avoid deleterious effects which could be a major obstacle for using such agents in certain clinical settings.

MCC950, one of the most promising inhibitors specifically blocking NLRP3 and thereby IL-1β and IL-18 activation, is already in clinical development [8,9]. MCC950 has been shown to be renoprotective in a preventive study of DKD using the db/db model of type 2 diabetes, however the aforementioned study did not investigate the renoprotective potential of MCC950 as an interventional study design in an established model of DKD or in models of type 1 diabetes [10]. From a clinical perspective, as diabetic subjects most often present with established renal disease, we aimed to investigate the renal impact of delayed intervention with MCC950 in mice with established DKD.

## Materials and methods

### Study design

In order to examine the effect of NLRP3 inflammasome inhibition by MCC950 [9] on renal pathology, 8-week-old male non-diabetic and diabetic *ApoE*^−/−^ mice with established DKD were treated with MCC950. We designed two separate studies of different durations to measure the short-term impact of MCC950 on renal gene expression which is affected early in diabetes as well as the long-term impact on kidney histology and protein expression which become more evident at a later stage. In both the studies, the impact of MCC950 was investigated by comparing the following groups, 1; control (C), 2; diabetes (D), 3; control+MCC950 (C+MCC950) and 4; diabetes+MCC950 (D+MCC950). In the shorter study to assess renal gene expression, MCC950 treatment was started after 5 weeks of diabetes and maintained throughout the remaining 5 weeks of the study, i.e. total duration was 10 weeks of diabetes. In the longer study to assess kidney histology and protein expression, MCC950 treatment was initiated after 9 weeks of diabetes and maintained for the remaining 9 weeks of the study, i.e. total duration of diabetes was 18 weeks. The studies were approved by the Alfred Medical Research and Education Precinct (AMREP) Animal Ethics Committee under guidelines laid down by the National Health and Medical Research Council of Australia. Animal experiments were performed in the laboratory at the Department of Diabetes, Central Clinical School, Monash University, Melbourne, Australia.

Diabetes was induced with streptozotocin (100 mg/kg intraperineally (i.p.), Sigma–Aldrich Corp, St. Louis, MO) dissolved in 0.1 M citrate buffer (pH 4.5) and injected on two consecutive days, whereas non-diabetic control animals were injected with citrate buffer only. MCC950 (kindly supplied by Prof. Avril Robertson, University of Queensland, Australia) was administered i.p. three times per week (5 mg/kg in 3% DMSO) whereas vehicle-treated animals were given 3% DMSO in PBS three times per week i.p. [9]. This is a dose of MCC950 previously shown to be vasoprotective, albeit renal effects were not reported in that study [11].

The animals were housed on wood granulate bedding in a pathogen-free environment with an artificial 12-h:12-h light/dark cycle with free access to standard chow (Specialty Feeds, Perth, WA, Australia) and water.

At the end of the study, the animals were killed using sodium pentobarbital (100 mg/kg body weight; Euthatal; Sigma–Aldrich, Castle Hill, NSW, Australia) and had blood drawn into heparinized sample tubes by right ventricle heart puncture for the separation of plasma. Right and left kidneys were subsequently dissected, weighed and fixed in 10% formalin and embedded in paraffin as well as fresh frozen in liquid nitrogen for storage at −80°C. Blood glucose and glycated hemoglobin were measured, as described previously [12,13]. Blood pressure was measured using a computerized non-invasive tail-cuff method as previously described on unanesthetized animals [14]. Mice with blood glucose ≥ 15 mmol/l have been included in the experiments; mice with blood glucose < 15 mmol/l and with polycystic kidneys were excluded from the study (<5% of the total number of mice).

### Kidney function and urinalysis

Plasma cystatin C, a marker of renal function, was quantified using a commercially available ELISA kit according to the manufacturer’s instructions (cat. no. RD291009200R, Biovendor, Brno, Czech Republic). Urine was collected by placing the animals individually in metabolic cages 1 week prior to the end of the study. Urinary albumin concentration was measured by ELISA quantification kit according to the manufacturer’s instructions (cat. no. E99-134, Bethyl Laboratories, Montgomery, TX) and urinary creatinine was measured using a creatinine kit on the Cobas Integra 400 Plus analyzer. Urinary 8-isoprostane, a marker of oxidative stress, was quantified using a commercially available kit (cat. no. 516351, Cayman Chemical, Ann Arbor, MI) and expressed normalized to urinary creatinine concentration.

The Quantikine Mouse MCP-1 ELISA kit (R&D Systems, Minneapolis, MN, U.S.A.) was used for the estimation of plasma monocyte chemoattractant protein-1 (MCP-1) levels, as per the kit instructions in mice from the long-term study.

### Kidney histology and immunohistochemistry

Four-micrometer-thick sections were cut from formalin-fixed paraffin embedded kidney preparations for histology and immunohistochemistry. Sections were stained with Periodic Acid–Schiff and digitalized for the measurement of mesangial volume as a percentage of total glomerulus using Image-Pro Plus 7 (Media Cybernetics, Bethesda, MD) as well as glomerulosclerosis index (GSI) and tubulointerstitial area as previously described [15,16]. In brief, glomerular injury including mesangial matrix accumulation, hyalinosis, capillary dilation and cellularity was graded on a scale from 0 to 4 corresponding to injury affecting an increasing fraction of the individual glomerulus, i.e., grade 0; no injury was seen, grade 1; 0–25% affected, grade 2; 25–50% affected, grade 3; 50–75 affected, grade 4; 75–100% affected whereas tubulointerstitial area was measured by a point-counting technique.

Immunohistochemistry was performed as previously described with minor modification [12]. In brief, sections were dewaxed and rehydrated, endogenous peroxidase was quenched using hydrogen peroxide and blocked using either normal horse serum or milk powder and Tween-20. The sections were then stained using antibodies either detecting collagen IV (1:450, cat. no. 1340-01 Southern Biotech, Birmingham, AL), fibronectin (1:1000, cat. no. A0245, Dako, Glostrup, Denmark), nitrotyrosine (1:350, cat. no. AB5411, Millipore, Billerica, MA), the macrophage marker CD68 (1:200, cat. no. ab125212, Abcam, Cambridge, U.K.) or NLRP3 (1:200, cat. no. AG-20B-0014-C100, Adipogen AG Liestal, Switzerland). Following incubation with the primary antibody, sections were washed and blocked with an avidin/biotin blocking kit (cat. no. SP-2001, Vector Laboratories, Burlingame, CA), incubated with a biotinylated secondary antibody after which a chromogen signal was developed with a peroxidase kit (cat. no. PK-6100, Vector Laboratories). Collagen IV and fibronectin deposition were evaluated as the percentage of the glomerulus that was positive whereas nitrotyrosine and NLRP3 were normalized to the level in the vehicle-treated control animals. CD68 staining was evaluated by counting the number of CD68-positive cells within the glomeruli. Histology and immunohistochemistry were evaluated based on 20 images per animal using Image-Pro Plus 7.

### Western blotting

Protein was extracted from snap-frozen renal cortex samples homogenized in lysis buffer containing a protease inhibitor cocktail (cat. no. B14012, Bimake, Houston, TX). Electrophoresis was performed under reducing conditions on a 4–20% polyacrylamide gel (cat. no. 4568096, Bio-Rad, Hercules, CA) and transferred to a PVDF membrane (cat. no. 1704156, Bio-Rad). The membrane was then blocked in 5% bovine serum albumin and incubated with primary antibodies against caspase-1 (1:1000, cat. no. ab179515, Cambridge, U.K.), IL-1β (1:1000, cat. no. AF-401-NA, R&D systems, Minneapolis, MN) or β-actin (1:5000, cat. no. 4970, Cell Signaling Technology, Danvers, MA). The target protein was subsequently detected using the signal from fluorophore-conjugated secondary antibodies read on an Odyssey CLX 1664 (LI-COR Biotechnology, Lincoln, NE) and expressed relative to β-actin.

### Gene expression

Snap-frozen samples of mouse renal cortex from short-term study were used for the extraction of RNA with Direct-zol RNA MiniPrep Kit (cat. no. R2052, Zymo Research, Irvine, CA) after homogenization in a Bullet Blender Storm 24 (Next Advance, Troy, NY). cDNA was synthesized using a reverse transcriptase kit (cat. no. BIO-65054, Bioline Meridian Life Science, Memphis, TN) which was used for the measurements of target gene expression relative to the housekeeping gene 18S (ABI Prism 7500, Perkin-Elmer, Poster City, CA) [12]. Probe and primer sequences used for qRT-PCR are shown in Supplementary Table S1.

### *In vitro* experiments

Conditionally immortalized human podocytes were used for *in vitro* experiments [17]. Podocytes were grown in RPMI with 10% FCS and ITS media supplement (Sigma–Aldrich) in a humidified incubator, 5% CO_2_ at 33°C. Cells were differentiated by transferring approximately 60% confluent cells to 2% FBS media and incubated at 37°C for 2 weeks. Cells were then grown in RPMI with 5 or 25 mmol/l glucose together primed with lipopolysaccharide (LPS, 0.5 µg/ml) [18] and incubated for 18 h at 37°C followed by 4 h of treatment with MCC950 (10 µM) [11]. Cells were harvested and RNA was extracted by the TRIzol method and cDNA was synthesized for quantitative RT-PCR as described previously [12]. Results were expressed relative to the untreated normal glucose control, which was arbitrarily assigned a value of 1. *In vitro* experiments were repeated three times. Human probe and primer sequences used for quantitative RT-PCR are shown in Supplementary Table S2.

### Statistical analysis

All data were analyzed by one-way ANOVA for multiple comparison of the means followed by Tukey’s post hoc test or analyzed by the two-tailed unpaired *t* test when required. Data were analyzed using GraphPad Prism 8 (San Diego, CA) and expressed as mean ± SEM. *P*<0.05 was considered as statistically significant. ns indicates not significant.

## Results

### Metabolic parameters

In both the short- and long-term studies, increased levels of blood glucose, HbA1c, water intake and urine production as well as decreased body weight were observed in both the vehicle-treated and the MCC950-treated diabetic animals compared with their respective controls (Table 1). Neither of these parameters differed in MCC950-treated diabetic animals as compared with vehicle-treated diabetic animals. Blood pressure did not vary among the groups (Table 1).

**Table 1:** Metabolic and kidney function measurements in diabetic and control animals receiving either MCC950 treatment or vehicle only

	Short-term study (10 weeks of diabetes)				Long-term study (18 weeks of diabetes)			
	Vehicle		MCC950 in week 5–10		Vehicle		MCC950 in week 9–18	
	Control	Diabetes	Control	Diabetes	Control	Diabetes	Control	Diabetes
Body weight (g)	30.8 ± 0.5	25.8 ± 1.1***	29.5 ± 0.5	25.9 ± 0.9**	31.7 ± 0.5	24.6 ± 1.4***	30.7 ± 1.0	26.3 ± 1.4**
Right kidney/BW (mg/g)	6.17 ± 0.15	7.3 ± 0.40**	6.49 ± 0.17	6.91 ± 0.29	6.7 ± 0.16	8.44 ± 0.44***	6.30 ± 0.13	7.95 ± 0.47***
Left kidney/BW (mg/g)	6.31 ± 0.16	7.20 ± 0.42*	6.31 ± 0.14	6.51 ± 0.20	6.8 ± 0.11	8.21 ± 0.39**	6.22 ± 0.13	8.09 ± 0.61***
P-glucose (mmol/l)	10.3 ± 0.4	25.7 ± 2.1***	10.2 ± 0.6	23.6 ± 1.2***	10.1 ± 0.4	30.6 ± 1.8***	11.6 ± 0.6	29.3 ± 1.7***
HbA1c (%)	4.3 ± 0.2	9.1 ± 0.6***	4.0 ± 0.1	8.1 ± 0.3***	4.1 ± 0.1	9.7 ± 1.1**	4.4 ± 0.2	9.3 ± 1.0**
Systolic BP (mmHg)	99 ± 4.6	101 ± 4.6	88 ± 6.7	92 ± 6.8	98 ± 3.8	100 ± 3.2	103.8 ± 6.2	98.5 ± 5.9
Food intake (g)	2.9 ± 0.2	3.7 ± 0.2	2.8 ± 0.2	4.0 ± 0.4**	2.6 ± 0.15	5.1 ± 0.3***	2.7 ± 0.3	4.3 ± 0.5***
Water intake (ml)	2.8 ± 0.6	10.1 ± 1.6***	2.1 ± 0.3	9.5 ± 2.9***	1.9 ± 0.3	17.0 ± 2.5***	1.8 ± 0.2	15.3 ± 3.2***
Urine output (ml)	1.06 ± 0.17	9.9 ± 1.5***	1.1 ± 0.14	8.7 ± 2.9***	0.85 ± 0.17	15.56 ± 2.13***	0.65 ± 0.14	13.69 ± 3.02***
Cystatin C (ng/ml)	300 ± 34	204 ± 19**	293 ± 24	224 ± 13*	230 ± 11	190 ± 12*	233 ± 13	176 ± 14**
ACR (mg/g)	45.1 ± 3.6	126.3 ± 17.5**	50.4 ± 3.1	104.4 ± 22.2*	30.2 ± 4.9	78.2 ± 13.8*	41.1 ± 6.5	160.5 ± 40.3**^#^

Data are presented as mean ± SEM. Abbreviations: ACR, urinary albumin-to-creatinine ratio; BP, blood pressure; BW; body weight; P; plasma. *n*=7–11 per group. Asterisks represent *P*-values for the comparisons of diabetes vs. control and diabetes+MCC950 vs. control+MCC950, respectively. *<0.05, **<0.01, *** <0.001. ^#^ represents *P*=0.06 for the comparison of diabetes+MCC950 vs untreated diabetes.

### Renal function

The urinary albumin-to-creatinine ratio (ACR) was increased in both diabetic groups compared with their respective control groups (Table 1). In the long-term study, we observed that MCC950-treated diabetic animals tended to have a higher ACR than the non-treated diabetic animals (*P*=0.06) (Table 1). Plasma cystatin C, as a measure of kidney function, was significantly lower in the diabetic groups as compared with their respective control groups consistent with diabetes associated hyperfiltration. Plasma cystatin C levels did not differ between the MCC950-treated and vehicle-treated diabetic animals (Table 1).

### Renal morphology

**Figure 1:**
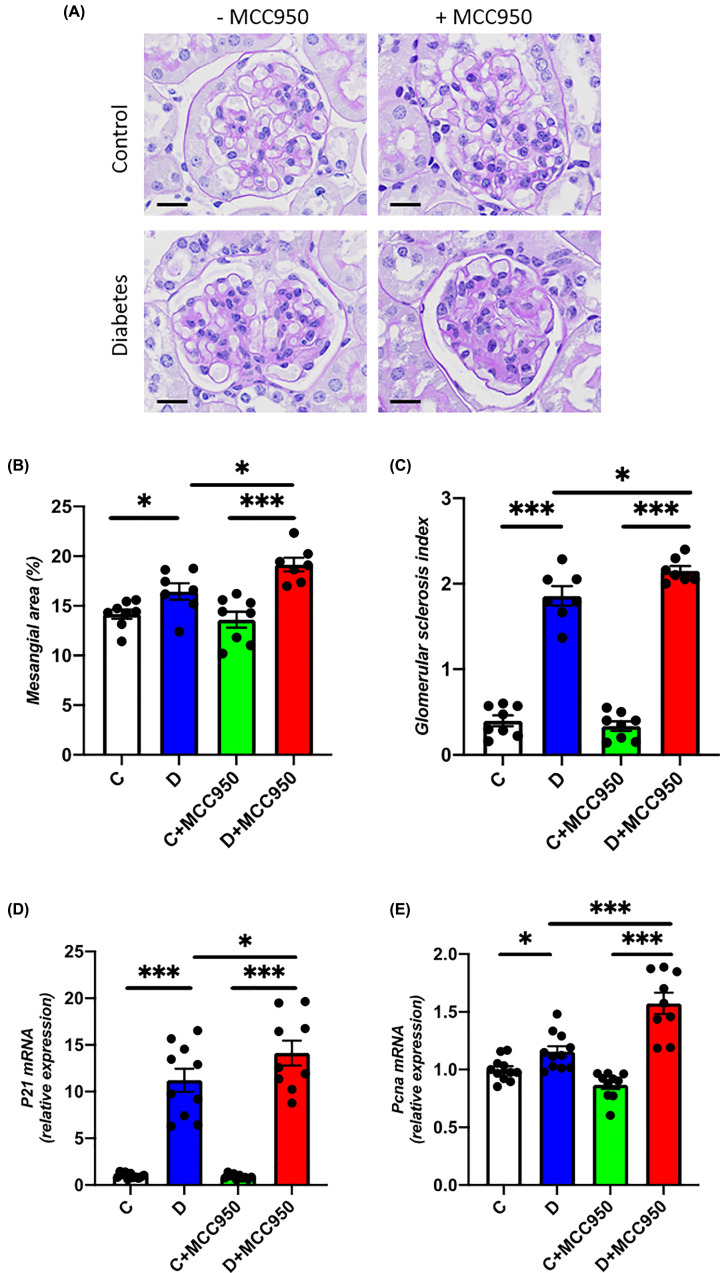
MCC950 increases renal injury in diabetic mice (**A**) Representative images of glomeruli sections stained with Periodic Acid–Schiff (scale bars indicating the length of 20 µm), (**B**) mesangial area per glomerulus and (**C**) GSI in the long-term study. Renal cortical gene expression of (**D**) *P21* and (**E**) *Pcna* in the short-term study (10 weeks). C, control; D, diabetes; C+MCC950, MCC950-treated control and D+MCC950, MCC950-treated diabetic mice. Asterisks represents *P*-values for comparisons of the indicated groups: *<0.05 and ***<0.001. *n*=7–11/group.

Renal hypertrophy was evaluated as kidney weight relative to body weight. The kidney to body weight ratio was increased in both diabetic groups as compared with their respective control groups in the long-term study (Table 1) and there were no differences in kidney to body weight ratio between MCC950-treated and vehicle-treated diabetic animals. The impact of MCC950 treatment on renal morphology in diabetes was subsequently assessed by measuring mesangial area and GSI (Figure 1A–C). Mesangial area and GSI were increased in both diabetic groups as compared with their respective control groups (Figure 1A–C). MCC950 treatment augmented the diabetes related structural injury as indicated by a significantly increased mesangial area and GSI in MCC950-treated compared with vehicle-treated diabetic animals (*P*<0.05). We also identified that the renal mRNA expression of the cell cycle regulator *P21* and the proliferation marker *Pcna* were up-regulated in both diabetes groups as compared with their respective controls (Figure 1D,E). Both genes were further up-regulated in the MCC950-treated diabetes group as compared with the vehicle-treated diabetes group.

### Renal inflammation

**Figure 2:**
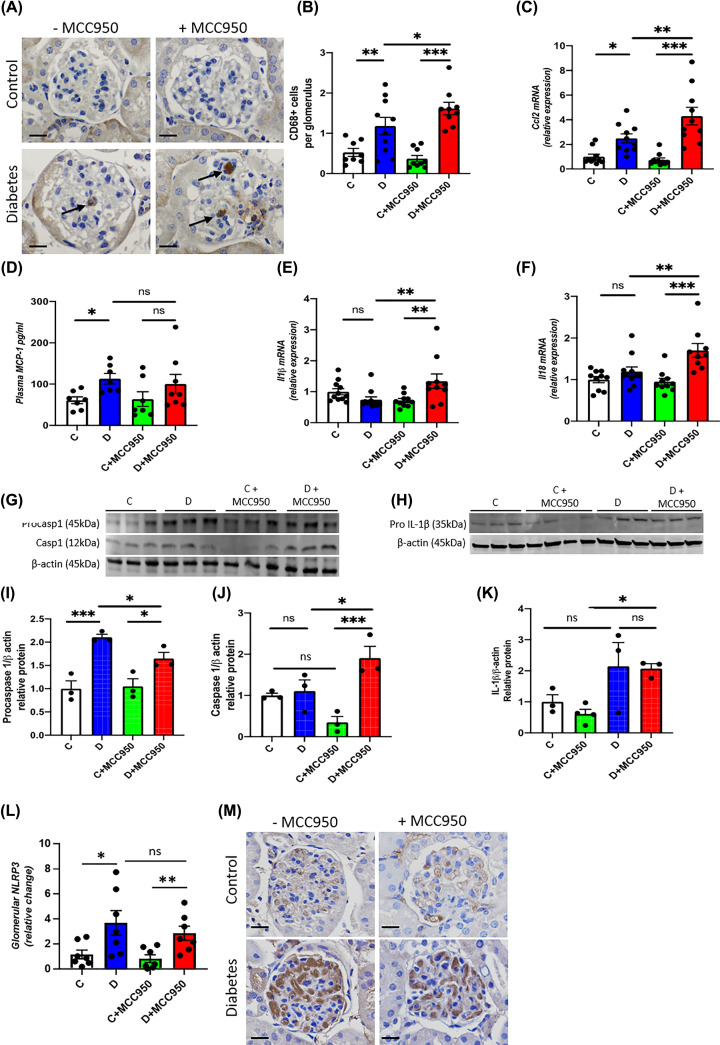
MCC950 enhances renal inflammation in diabetic mice (**A**) Representative images of immunohistochemistry targeting CD68 (scale bars 20 µm and the arrows indicating CD68-positive cells), (**B**) quantification as number of CD68 positive cells per glomerulus and (**D**) plasma concentration of MCP-1 in the long-term study. Renal cortical gene expression of (**C**) *Ccl2* also known as MCP-1, (**E**) *Il1β* and (**F**) *Il18* in the short-term study. Western blot on renal cortex of procaspase 1 (45 kDa) and caspase 1 (12 kDa) (**G**) as well as pro-IL-1β (35 kDa) (**H**) and their quantification (**I**) procasp1 (procaspase 1), (**J**) casp1 (caspase 1) and (**K**) pro-IL-1β relative to β-actin (45 kDa) in the long-term study. (**L**,**M**) Glomerular protein expression of NLRP3 and representative images of immunohistochemistry targeting NLRP3 (scale bars 20 µm). C, control; D, diabetes; C+MCC950, MCC950-treated control and D+MCC950, MCC950-treated diabetic mice. Asterisks represent *P*-values for comparisons of the indicated groups: *<0.05, **<0.01 and *** <0.001. *n*=7–11/group for panels (A–F,L–M), *n*=3–4/group for panels (G–K) (Western blot).

The diabetic groups had significantly more CD68 positive cells within the glomeruli than their respective control groups (Figure 2A,B). The MCC950-treated diabetic animals demonstrated a significantly increased macrophage infiltration within the glomeruli as compared with the vehicle-treated diabetic animals, suggestive of enhanced inflammation induced by MCC950 treatment in diabetes (Figure 2A,B). Consistent with macrophage infiltration, the mRNA level of renal *Ccl2* which encodes for *MCP-1* was further increased in MCC950-treated diabetic animals (Figure 2C). In addition, we subsequently measured MCP-1 in plasma taken at the end of the long-term study in order to quantify the systemic effect of long-term MCC950 treatment. The vehicle-treated diabetic animals had higher plasma MCP-1 levels compared with control group. Interestingly, no statistically significant difference was observed between MCC950-treated diabetic and control group (Figure 2D). Moreover, no significant difference was observed between the MCC950-treated and vehicle diabetic groups.

The renal mRNA levels of the inflammatory markers *Il1β* and *Il18* did not differ significantly between vehicle-treated diabetic and control animals (Figure 2E,F). However, both these markers were up-regulated in MCC950-treated diabetic animals compared with MCC-treated controls and with vehicle-treated diabetic animals (Figure 2E,F).

The renal expression of procaspase 1, a component of the NLRP3 inflammasome was increased in both groups of diabetic mice in comparison to their respective non-diabetic controls (Figure 2G,I). Diabetic mice treated with MCC950 showed a lower level of procaspase 1 in the kidney when compared with untreated diabetic mice (Figure 2G,I). On the other hand, the renal protein expression of caspase-1 was increased in diabetic mice treated with MCC950 in comparison to non-diabetic counterparts as well as with vehicle-treated diabetic mice (Figure 2G,J). The protein level of pro-IL-1β was higher in the MCC950-treated diabetic group compared with the treated controls (Figure 2H,K). The same pattern was seen in the non-treated diabetic group compared with the controls, but the difference did not reach to statistical significance (*P*=0.07). Pro-IL-1β levels did not differ between the two diabetic groups (Figure 2H,K). Moreover, renal expression of NLRP3 was found to be increased in both groups of diabetic mice (Figure 2L,M). However, no significant difference was observed between vehicle or MCC950-treated diabetic mice (Figure 2L,M). To complement the *in vivo* findings, we examined the effect of MCC950 in human podocytes exposed to high glucose and LPS. Expression of both *NLRP3* and *IL-1β* were increased in high glucose- and LPS-treated human podocytes (Figure 5A,B). However, high glucose- and LPS-induced up-regulation of *NLRP3* and *IL-1β* were not significantly attenuated by MCC950 treatment (Figure 5A,B). Expression of IL-18 remained unchanged (Figure 5C). Interestingly, treatment with MCC950 led to further up-regulation of *MCP-1* and *TLR4* in human podocytes exposed to high glucose and LPS (Figure 5D,E).

### Renal oxidative stress

**Figure 3:**
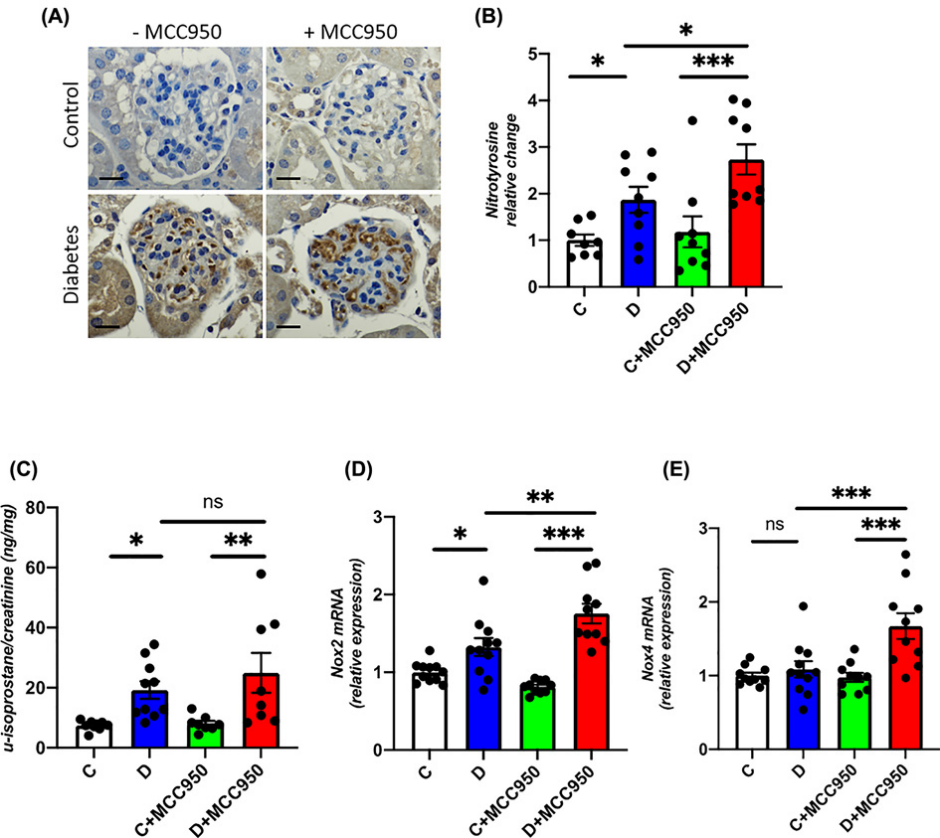
MCC950 enhances renal oxidative stress in diabetic mice (**A**) Representative images of immunohistochemistry targeting nitrotyrosine (scale bars 20 µm) and (**B**) its quantification within the glomeruli as well as (**C**) urinary isoprostane relative to urinary creatinine in the long-term study. Renal cortical gene expression of (**D**) *Nox2* and (**E**) *Nox4* in the short-term study. C, control; D, diabetes; C+MCC950, MCC950-treated control and D+MCC950, MCC950-treated diabetic mice. Asterisks represent *P*-values for comparisons of the indicated groups: *<0.05, **<0.01 and *** <0.001. *n*=8–11/group.

Glomerular oxidative stress as measured by nitrotyrosine immunohistochemistry was increased in both diabetic groups compared with their respective control group (Figure 3A,B). In addition, the glomerular nitrotyrosine content was further increased in the MCC950-treated diabetic group compared with the vehicle-treated diabetic group suggesting enhanced production of reactive oxygen species (ROS) with MCC950 treatment. The urinary excretion of 8-isoprostane normalized to urinary creatinine was quantified as a measure of systemic oxidative stress. Both diabetic groups had higher urinary 8-isoprostane excretion compared with their respective control groups, but no difference was seen between the MCC950-treated and non-treated diabetic groups (Figure 3C). In addition, vehicle-treated diabetic kidneys had increased gene expression of *Nox2* but not *Nox4* (Figure 3D,E). However, MCC950-treated diabetic animals had significantly higher renal expression of both Nox2 and Nox4 compared with the vehicle-diabetic animals (Figure 3D,E). Similar to MCC950-treated diabetic animals, high glucose- and LPS-treated human podocytes showed further up-regulation of pro-oxidant factors *NOX4* and *NOX2* (Figure 5F,G).

### Renal fibrosis

**Figure 4:**
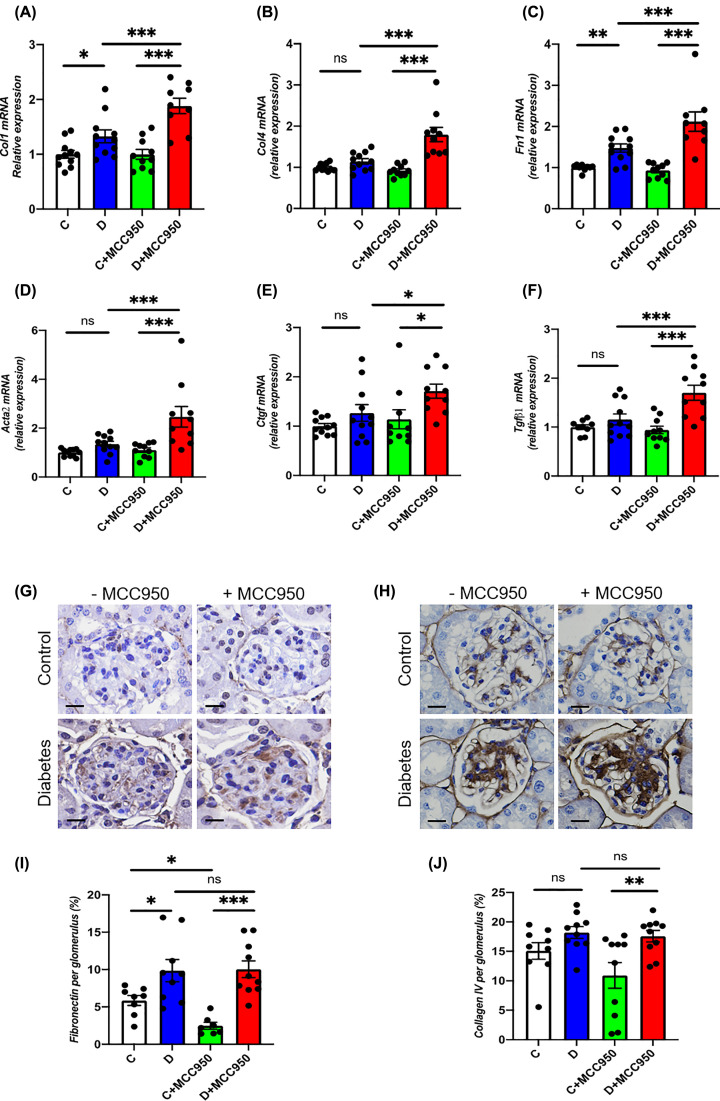
MCC950 promotes up-regulation of pro-fibrotic factors in DKD Renal cortical gene expression of (**A**) *Col1*; collagen 1, (**B**) *Col4*; collagen 4, (**C**) *Fn1*; fibronectin (**D**) *Acta2*; Actin α 2 also known as α-SMA, (**E**) *Ctgf* and (**F**) *Tgfβ1* in the short-term study. Representative images of immunohistochemistry targeting fibronectin (**G**) and Collagen 4 (**H**) and their quantification (**I,J**) respectively within the glomeruli (scale bars 20 µm) in the long-term study. C, control; D, diabetes; C+MCC950, MCC950-treated control and D+MCC950, MCC950-treated diabetic mice. Asterisks represent *P*-values for comparisons of the indicated groups: *<0.05, **<0.01 and *** <0.001. *n*=7–11/group.

In-line with the observed adverse effects of MCC950 on renal morphology, multiple markers of renal fibrosis were up-regulated by MCC950 treatment in diabetes. Indeed, renal gene expression of the extracellular matrix proteins, C*ol1*, *Col4* and *Fn1* (fibronectin) was up-regulated in both diabetic groups as compared with their respective non-diabetic control groups with a further increase in MCC950-treated diabetic animals in comparison to the vehicle-treated diabetic animals (Figure 4A–C). In addition, renal gene expression of *Acta2* (α-smooth muscle actin, α-SMA), *Ctgf* and *Tgfβ* was up-regulated in MCC950-treated diabetic animals compared with MCC950-treated controls (Figure 4D–F). Importantly, the gene expression of these fibrotic markers was significantly higher in the MCC950-treated diabetic animals as compared with the vehicle-treated diabetic animals (Figure 4D–F). Furthermore, glomerular accumulation of fibronectin was increased in both diabetic groups as compared with respective controls animals (Figure 4G,I). For collagen IV, the MCC950-treated diabetic mice showed a significantly higher collagen IV accumulation compared with control MCC950-treated mice (Figure 4H,J). However, no difference was observed in collagen IV and fibronectin staining when comparing the MCC950-treated diabetic animals with the vehicle-treated diabetic groups. MCC950-treated control animals had a lower fibronectin content than the vehicle-treated control animals similar to the trend observed for collagen IV accumulation (Figure 4G–J).

**Figure 5:**
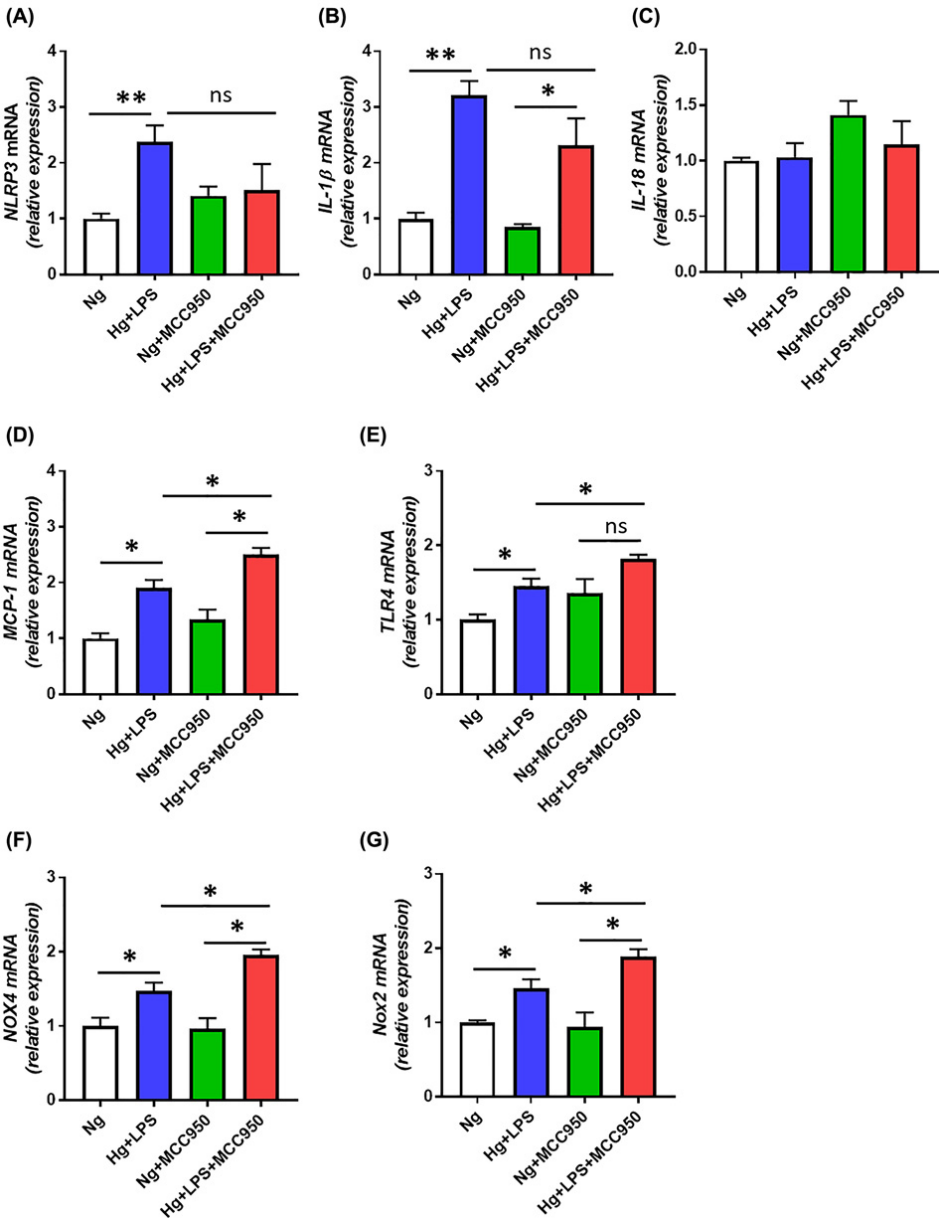
Effect of MCC950 on markers of inflammation and oxidative stress in human podocytes exposed to glucose and LPS (**A–E**) Gene expression of markers of inflammation *NLRP3* (A), *IL-1β* (B), *IL-18* (C), *MCP-1* (D), TLR-4 (E); (**F,G**) gene expression of pro-oxidant factors NOX4 (F) and NOX2 (G) in human podocytes in response to normal glucose (NG, 5 mM) and high glucose (HG, 25 mM) primed with LPS (0.5 µg/ml) with and without MCC950 (10 µM). Data are shown as mean ± SEM. Asterisks represent *P*-values for comparisons of the indicated groups: * <0.05, ** <0.01, and ns, not significant statistically.

## Discussion

Inhibition of the NLRP3 inflammasome is attracting increasing attention as a new drug target since it has been reported to be involved in the pathogenesis of multiple diseases including DKD [2–5]. The present study however, provides substantial evidence to indicate that one must be cautious in assuming a beneficial impact of NLRP3 inflammasome inhibitors on various organs as our results have failed to show a renoprotective effect of MCC950, while additionally indicating potential adverse effects on the kidney in an established model of DKD. Indeed, diabetic animals treated with MCC950 were found to have enhanced mesangial expansion and increased glomerulosclerosis as compared with vehicle-treated diabetic animals. In addition, up-regulation of cell cycle regulator *p21* [19] and cell proliferation marker *Pcna* [20] have been shown to be associated with enhanced mesangial expansion and glomerulosclerosis in DKD [21]. Consistent with enhanced mesangial expansion, further up-regulation of *p21* and *Pcna* were found in MCC950-treated diabetic animals. Furthermore, we observed an increased renal gene expression of the ROS-producing NADPH oxidases *Nox2* and *Nox4* and a higher glomerular content of nitrotyrosine in diabetic animals treated with MCC950 compared with vehicle-treated diabetic animals. Similarly, renal gene expression of *Il1β*, *Il18* and *Ccl2* as well as glomerular accumulation of CD68-positive cells, which is a macrophage marker, were increased in the MCC950-treated diabetic animals, consistent with MCC950 promoting renal inflammation in diabetes. In addition, treatment of MCC950 led to further up-regulation of markers of inflammation and oxidative stress in human podocytes exposed to high glucose and LPS, supporting an association between inflammation and oxidative stress in DKD.

A substantial body of evidence has demonstrated a contribution of inflammation to the development of DKD [22]. Indeed, it has been shown that diabetic animals have an increased expression of various components of the NLRP3 inflammasome as well as increased caspase 1 activation and IL-1β and IL-18 maturation [23,24]. In line with these findings, Shahzad and colleagues report on human data demonstrated an increased expression of NLPR3 protein in the glomeruli of type 2 diabetic patients with DKD as opposed to diabetic patients without DKD and non-diabetic control subjects [5]. Furthermore, the author reported that genetic knockout of either NLPR3 or caspase-1 improved urinary albumin excretion and mesangial expansion in uninephrectomized streptozotocin-diabetic mice, an accelerated model of DKD [5]. However, one must be cautious in extrapolating findings in that model to diabetes *per se* since uninephrectomy is likely to exacerbate renal injury in a hemodynamic yet non-glucose dependent manner. Still, it is striking that MCC950 treatment of diabetic mice in our study led to an increase in renal caspase 1 protein compared with vehicle-treated diabetic animals although in an STZ-induced diabetic model. In addition, our data demonstrate that diabetes induced increased expression of NLRP3 inflammasome components pro-caspase 1 and NLRP3 were not significantly attenuated by MCC950. Shahzad and colleagues also demonstrated that inhibiting the IL-1 receptor by Anakinra thereby blocking one of the effector molecules of NLRP3 inflammasome activation, IL-1β, prevented or even reversed diabetes associated albuminuria and mesangial expansion in streptozotocin diabetic and db/db diabetic mice [5]. In additional experiments using bone marrow transplantation from either NLPR3 or caspase-1 deficient mice to wildtype mice and *vice versa* suggested that the NLPR3 inflammasome-induced albuminuria and mesangial expansion as seen in diabetic animals is caused by cells resident within the kidney [5].

On the basis of these reports implicating the NLRP3 inflammasome in DKD, it is of particular interest to explore the efficacy of NLRP3 inhibition using a highly specific compound, MCC950, that specifically targets the ATP hydrolysis motif of the NLRP3 protein thereby inhibiting assembly of the inflammasome complex [8,9,25]. The impact of MCC950 as primary prevention on the development of DKD has recently been studied by Zhang and colleagues in a model of type 2 diabetes [10]. In that study of db/db mice that develop diabetes because of leptin receptor mutation-induced hyperphagia with associated obesity and hyperinsulinemia and later hypoinsulinemia, 10 mg MCC950 per kg body weight was administered i.p. twice per week for 12 weeks. The authors reported that MCC950 treatment improved diabetes-induced kidney injury on multiple parameters including serum creatinine, urinary albumin excretion, mesangial expansion and the renal gene and protein expression of mediators of kidney injury including TGF-β, collagen 1, fibronectin and α-SMA. Also, in their study MCC950-treatment led to a decrease in the renal caspase 1 protein level in contrast with our study in which streptozotocin diabetic animals given MCC950 treatment had a higher caspase 1 level with enhanced renal inflammation and oxidative stress.

Indeed, our results show aggravation or no benefit of MCC950 treatment for many of the pathophysiological features of DKD. A possible explanation for these discordant results may reside in the different mouse models of diabetes that were investigated. Zhang and colleagues model is a spontaneous type 2 model of insulin resistance while our model is more consistent with insulin-deficient type 1 diabetes in *ApoE*^−/−^ mice [10]. This may explain the major dissimilarities in terms of food intake, body mass and composition and insulin levels [10]. Their study design also differs from our study which is prevention versus an interventional approach in an established model of DKD. Zhang and colleagues used the former approach, where prevention of DKD was investigated with MCC950 treatment initiated at baseline, while in our study an interventional strategy was used where MCC950 treatment was delayed until DKD was established with the aim of improving the disease or preventing further deterioration. Our interventional study therefore more closely mimics the clinical situation where most patients present with established DKD before treatment.

The two studies also differ slightly with respect to the MCC950 dose regimen, i.e., a total weekly dose of 15 mg/kg in our study vs. 20 mg/kg by Zhang and colleagues [10]. However, the difference is nominal and unlikely to have contributed to these discordant results. Furthermore, the previous study [10] also differs from our design in that they did not include an MCC950-treated non-diabetic group to distinguish the effect of this drug in diabetes-specific versus independent of diabetes. Comparing our findings with those of Shahzad and colleagues are more complicated as their study involved genetic knockout of essential components of the NLRP3 inflammasome, *Nlrp3* and *Casp1*, to study the impact of the inflammasome as opposed to a pharmacological inhibitor [5].

MCC950 has previously been shown to be effective in many inflammatory diseases, including hypertensive kidney disease [26] and is in clinical development for Parkinson’s disease. However, it surprisingly failed to be effective on β-cells in db/db mice where low-grade inflammation is prominent indicating discordant effects of blocking NLRP3 inflammasome activation [27]. We now show no protection of MCC950 in the diabetic kidney despite a clear reduction in vascular inflammation and atherosclerosis as recently demonstrated by our group at the same dose of MCC950 in the same diabetic model [11]. In diabetes where renal and cardiovascular disease including atherosclerosis often coexist, preferred treatments are beneficial to both the vascular system and the kidney. Such examples are RAS inhibitors, mineralocorticoid receptor antagonists and more recently SGLT2 inhibitors. The present study has identified that the dose that is atheroprotective was not renoprotective and potentially has major implications for diabetic complications.

This implies that there may be organ-specific effects of inflammasome inhibition, as is suggested on reviewing the cardiovascular versus renal outcomes of patients in the CANTOS trial [6,7]. Even though in general the data indicate that it would be beneficial to block the NLRP3 inflammasome, the CANTOS trial may have unmasked potential adverse effects as reflected by the worsening of kidney function among subjects with chronic kidney disease in particular in established disease [7]. Although these observations were interpreted as clinically unimportant, this issue may be underappreciated and warrant further investigation. In conclusion, our results call for detailed evaluation of NLRP3 inflammasome blockers before assuming that they are end-organ protective in settings of low-grade inflammation such as in diabetic complications.

## Clinical perspectives

- Increasing evidence support the concept that NLRP3 inflammasome activation contributes to acute and chronic kidney disease including DKD.
- The present study reveals for the first time that NLRP3 inhibition with MCC950 did not show renoprotective effects in a model of established DKD. On the contrary, inhibition of NLRP3 by MCC950 in diabetic animals exhibited adverse renal effects including enhanced inflammation, oxidative stress and glomerulosclerosis.
- Our results therefore call for detailed evaluation of NLRP3 inflammasome blockers before assuming that they are end-organ protective in settings of low-grade inflammation such as in DKD.

## Supplementary Material

Supplementary Tables S1-S2

## Data Availability

The data underlying this article will be shared on reasonable request to the corresponding author.

## Open Access

Open access for this article was enabled by the participation of Monash University in an all-inclusive *Read & Publish* pilot with Portland Press and the Biochemical Society under a transformative agreement with CAUL.

## Abbreviations

ACR: urinary albumin-to-creatinine ratio
DKD: diabetic kidney disease
GSI: glomerulosclerosis index
LPS: lipopolysaccharide
MCP-1: monocyte chemoattractant protein-1
NLRP3: nucleotide-binding oligomerization domain-like receptor pyrin domain containing 3
ROS: reactive oxygen species
α-SMA: α-smooth muscle actin
