# Supplementary material for: Adverse renal effects of NLRP3 inflammasome inhibition by MCC950 in an interventional model of diabetic kidney disease
Source: Clin Sci (Lond). 2022 Jan 20;136(2):167–80. doi: 10.1042/CS20210865 (PMC8777085; doi:10.1042/CS20210865)

Supplemental table 1. Mouse probe and primer sequences for qRT-PCR

Genes	Probe Sequence (5'FAM-3" TAMRA)	Forward Primer 5'-3'	Reverse Primer 5'-3'
Nox2	CAACTGGACAGGAACCT	AGTGCGTGTTGCTCGACAAG	CCAAGCTACCATCTTATGGAAAGT
Nox4	CATTTTGCTATTTCATCAAA	AAAAATATCACACACTGAATTCGAGACT	TGGGTCCACAGCAGAAAACTC
MCP-1	AATGGGTCCAGACATAC	GTCTGTGCTGACCCCAAGAAG	TGGTTCCGATCCAGGTTTTTA
Collagen I	ATCGACCCTAACCAAG	GACTGGAAGAGCGGAGAGTACTG	CCTTGATGGCGTCCAGGTT
Collagen IV	CAGTGCCCTAACGGT	GGCGGTACACAGTCAGACCAT	GGAATAGCCGATCCACAGTGA
Fibronectin	CCCCGTCAGGCTTA	ACATGGCTTTAGGCGGACAA	ACATTCGGCAGGTATGGTCTTG
α-SMA	TGCCAGATCTTTTCC	GACGCTGAAGTATCCGATAGAACA	GGCCACACGAAGCTCGTTAT
Il1β	CTGAAAGCTCTCCACCTC	TCGTGCTGTCGGACCCATA	CTTGTACAAAGCTCATGGAGAATATCAC
Il18	SYBER	CACATGCGCCTTGTGATGAC	TGCAGCCTCGGGTATTCTGT
Tgf β1	AAAGCCCTGTATTCCGT	GCAGTGGCTGAACCAAGGA	GCAGTGAGCGCTGAATCGA
Ctgf	ACTGCCTGGTCCAGAC	GCTGCCTACCGACTGGAAGA	CTTAGAACAGGCGCTCCACTCT
Pcna	CACAGCTGTACTCCTGTTC	TCAAGAGAAAGTTTCAGACTATGAAATGA	AAATTCACCAGATGGCATCTTTATT
P21	AGAGCCACAGGCACC	TCCACAGCGATATCCAGACATT	CGGACATCACCAGGATTGG

Supplemental table 2: Human probes and primers for RT-PCR

Genes	Probe Sequence (6-FAM 5’-3’)	Forward Primer 5’-3’	Reverse Primer 5’-3’
NOX2	CCTCCTGCCATGACT	AGAGGGTTGGAGGTGGAGAATT	GCACAAGGAGCAGGACTAGATGA
NOX4	TCCATTTGCATCAATACT	GGCTGGAGGCATTGGAGTAA	CCAGTCATCCAACAGGGTGTT
MCP-1	CAGGAAACCAATATCCA	CAAAGCAGGGCTCGAGTTG	CCTGGGACTAGACTTGATGTCTCA
TLR-4	ATCCATGAAGGTTTCC	GGCCATTGCTGCCAACAT	CAACAATCACCTTTCGGCTTTT
NLRP3	CTGCGGACTGTCCCAT	GACCATCGGCCGGACTAAA	CGTCCTCGGGCTCAAACA
IL1β	SYBER	TTGGTGATGTGGTCCATATGA	TACAAAGGACATGGAGAACACCAC
IL18	CAGGAAATACGAAATGGA	TGTGTGATCGCCACAAACCT	CACTGCCCCGTATCTCAGAAA

Uncropped (original) gel used for western blot in figure. 2

Procasp1 (45kDa) and Casp1 (12kDa) – same gel

C D C + MCC950 D + MCC950

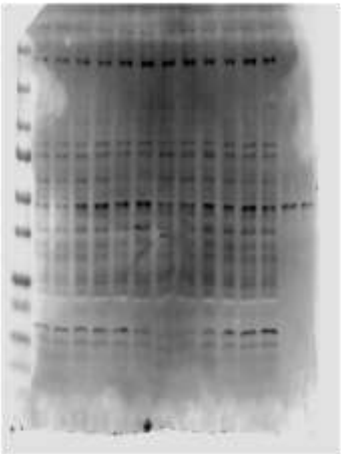

Pro IL-1β (35kDa)

C C + MCC950 D D + MCC950

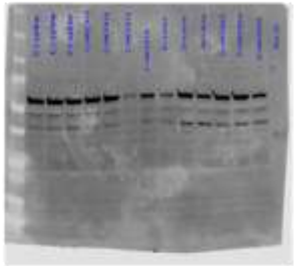

β-actin (45kDa) on gel for procasp1 and casp1

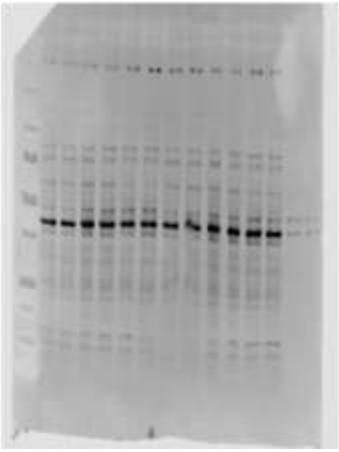

β-actin (45kDa) on gel for Pro IL 1beta

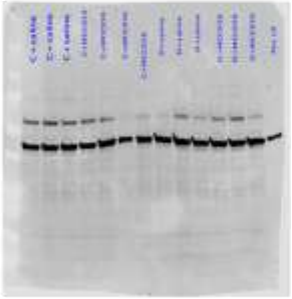

Supplement: Supplementary Tables S1-S2 [file CS-2021-0865_supp.pdf]
